# Supplementary material for: Social and Behavioral Determinants of Health in the Era of Artificial Intelligence with Electronic Health Records: A Scoping Review
Source: Health Data Sci. 2021 Aug 24;2021:9759016. doi: 10.34133/2021/9759016 (PMC10880156; doi:10.34133/2021/9759016)
Supplement: Supplementary Materials — Supplemental Document 1: literature searching strategies on Ovid, Scopus, Web of Science, ACM digital library, and IEEE Xplore. Supplementary Table 1: information extracted from the included studies. [file 9759016.f1.zip › Raw File_ Supplement.pdf]

Raw File: Social and behavioral determinants of health in the era of artificial intelligence with electronic health records: A scoping review

Citation	Country	SBDH Data Source	Clinical Note Type	Disease	Disease Category (ICD 10)	SBDH Focus	SBDH Category	SBDH level (individual, neighborhood)	SBDH Role	Study Cohort - #Patients	Study Cohort - #Clinical Sites	AI methods
H. M. Kim et al., "Predictors of suicide in patient charts among patients with depression in the Veterans Health Administration health system: importance of prescription drug and alcohol abuse," J. Clin. Psychiatry, vol. 73, no. 10, pp. e1269-75, Oct. 2012, doi: https://dx.doi.org/10.4088/JCP.12m07658.	USA	Structured and Unstructured Data	Progress notes/Admission notes/Discharge summaries	Depression	Mental, Behavioral and Neurodevelopmental disorders	Substance Use/Abuse	Health and Health Care	Individual	Contributed	636	Not specified	* Predictive modeling (logistic regression model)<br>* Statistical methods (bivariate analysis, multivariate analysis)
B. L. Hazlehurst et al., "Automating assessment of lifestyle counseling in electronic health records," Am. J. Prev. Med., vol. 46, no. 5, pp. 457–64, May 2014, doi: https://dx.doi.org/10.1016/j.amepre.2014.01.001.	USA	Unstructured Data	Progress notes/Primary care notes	Gestational diabetes mellitus (GDM)	Pregnancy, childbirth and the puerperium	Physical Activity; Diet	Health and Health Care	Individual	Contributed	500	2	* NLP (information extraction, Automated NLP-based processor (MediClass))
C. Dillahunt-Aspillaga, D. Finch, J. Massengale, T. Kretzmer, S. L. Luther, and J. A. McCart, "Using information from the electronic health record to improve measurement of unemployment in service members and veterans with mTBI and post-deployment stress," PLoS ONE Electron. Resour., vol. 9, no. 12, p. e115873, 2014, doi: https://dx.doi.org/10.1371/journal.pone.0115873.	USA	Structured and Unstructured Data	Progress notes	mild traumatic brain injury (mTBI) and post-deployment stress	Injury, poisoning and certain other consequences of external causes	Employment Status; Vocational History; Work-related Challenges	Economic Stability	Individual	Contributed	177	1	* NLP (annotation schema)
M. Richard, X. Aime, M.-O. Krebs, and J. Charlet, "Enrich classifications in psychiatry with textual data: an ontology for psychiatry including social concepts," Stud. Health Technol. Inform., vol. 210, pp. 221–3, 2015.	France	Unstructured Data	Discharges Summaries	Mental disorders (not a specific disease)	Mental, Behavioral and Neurodevelopmental disorders	Social Environment; Education	Social and Community Context; Education	Individual and Neighborhood	No mention	Not specified	1	* NLP (information exctraction, MELT, YATEA, MEDINA, DSM, ICD, SNOMED, FFH)
M. Poulymenopoulou, D. Papakonstantinou, F. Malamateniou, and G. Vassilacopoulos, "A health analytics semantic ETL service for obesity surveillance," Stud. Health Technol. Inform., vol. 210, pp. 840–4, 2015.	Greece	Structured and Unstructured Data	Progress notes/Primary care notes/Admission notes/Discharge summaries/Operative notes	Obesity	Endocrine, nutritional and metabolic diseases	Physical Activity; Diet; Sleep	Health and Health Care	Individual	No mention	Not specified	Not specified	* NLP (information exctraction, Protege OWL editor, Semantic Web Rule Language (SWRL), Jess rule engine, SPARQL)<br>* Predictive modeling (Apache Mahout)<br>* Other (Semantic ETL service, MongoDB and HBase, RestFull technology, Semantic ETL)
C. Nau et al., "Exploring the forest instead of the trees: An innovative method for defining obesogenic and obesoprotective environments," Health Place, vol. 35, pp. 136–146, Sep. 2015, doi: http://dx.doi.org/10.1016/j.healthplace.2015.08.002.	USA	Structured and Unstructured Data	Progress notes	Childhood obesity	Endocrine, nutritional and metabolic diseases	Geographic Location; Diet; Social Activity; Physical Activity; Social Charecteristics; Land Use Patterns	Neighborhood and Built Environment; Health and Health Care; Social and Community Context	Individual and Neighborhood	Contributed	22497	1	* Predictive modeling (Conditional Random Forests (CRF))
G. S. Kerr et al., "Measuring physician adherence with gout quality indicators: a role for natural language processing," Arthritis Care Res., vol. 67, no. 2, pp. 273–9, Feb. 2015, doi: https://dx.doi.org/10.1002/acr.22406.	USA	Structured and Unstructured Data	Progress notes/Primary care notes	Gout	Diseases of the musculoskeletal system and connective tissue	Diet; Substance Use/Abuse	Health and Health Care	Individual	Contributed	2,280	1	* NLP (information exctraction, Leo)<br>* Statistical methods (chi-square)
J. P. Anderson et al., "Reverse Engineering and Evaluation of Prediction Models for Progression to Type 2 Diabetes: An Application of Machine Learning Using Electronic Health Records," J. Diabetes Sci. Technol., vol. 10, no. 1, pp. 6–18, Dec. 2015, doi: https://dx.doi.org/10.1177/1932296815620200.	USA	Structured Data	NA	Diabetes	Endocrine, nutritional and metabolic diseases	Socioeconomic Factors	Economic Stability	Individual	Contributed	24,331	Database	* Predictive modeling (ensembles Using REFS, Kaplan–Meier plots, multivariable Cox regression models)
H. Zhang, N. Hosomura, M. Shubina, D. C. Simonson, M. A. Testa, and A. Turchin, "Electronic documentation of lifestyle counseling in primary care is associated with lower risk of cardiovascular events in patients with diabetes," Diabetes, vol. 65 (Supplement 1), p. A363, 2016, doi: http://dx.doi.org/10.2337/db16-1375-1656.	USA	Unstructured Data	NA	Diabetes	Endocrine, nutritional and metabolic diseases	Lifestyle	Health and Health Care	Individual	Contributed	10,870	2	* NLP (information exctraction)<br>* Statistical methods (multivariable Cox proportional hazards model), Levenstein distance
L. Tong, C. Erdmann, M. Daldalian, J. Li, and T. Esposito, "Comparison of predictive modeling approaches for 30-day all-cause non-elective readmission risk," BMC Med. Res. Methodol., vol. 16, p. 26, Feb. 2016, doi: https://dx.doi.org/10.1186/s12874-016-0128-0.	USA	Structured Data	NA	30-day all-cause nonelective readmission	Admission/Readmission	Employment Status; Substance Use/Abuse	Economic Stability; Health and Health Care	Individual	Minimal contribution	109,421	8	* Predictive modeling (LACE, STEPWISE, LASSO, AdaBoost)
S. Biro et al., "Utility of linking primary care electronic medical records with Canadian census data to study the determinants of chronic disease: an example based on socioeconomic status and obesity," BMC Med. Inform. Decis. Mak., vol. 16, p. 32, Mar. 2016, doi: https://dx.doi.org/10.1186/s12911-016-0272-9.	Canada	Structured Data	NA	Chronic diseases ; Obesity	Endocrine, nutritional and metabolic diseases	Socioeconomic Status (SES); Material and Social Deprivation; Socio-environmental Neighbourhood	Economic Stability; Social and Community Context; Neighborhood and Built Environment	Individual and Neighborhood	Contributed	7,186	1	* Statistical methods (descriptive statistics)
D. Agrawal et al., "Predicting patients at risk for 3-day postdischarge readmissions, ED Visits, and Deaths," Med. Care, vol. 54, no. 11, pp. 1017–1023, Oct. 2016, doi: http://dx.doi.org/10.1097/MLR.0000000000000574.	USA	Structured Data	NA	3-Day Postdischarge Readmissions	Admission/Readmission	Substance Use/Abuse; Socioeconomic Factors	Health and Health Care; Economic Stability	Individual and Neighborhood	No mention	64,952	5	* Predictive modeling (LR, random forests (RF), gradient boosting method, stacked generalization (stacking))
S. J. Patel, D. Chamberlain, and J. M. Chamberlain, "A machine-learning approach to predicting need for hospitalization for pediatric asthma exacerbation at the time of emergency department triage," Pediatr. Conf. Natl. Conf. Educ., vol. 142, no. 1, 2017, doi: http://dx.doi.org/10.1542/peds.142.1_MeetingAbstract.116.	USA, UK	Structured Data	NA	Pediatric Asthma	Diseases of the respiratory system	Socioeconomic Status (SES); Weather Features	Economic Stability; Neighborhood and Built Environment	Individual and Neighborhood	Contributed	29,392	2	* Predictive modeling (decision trees (DT), LASSO logistic regression, random forests (RF), gradient boosting machines)
E. A. Lindemann, E. S. Chen, Y. Wang, S. J. Skube, and G. B. Melton, "Representation of Social History Factors Across Age Groups: A Topic Analysis of Free-Text Social Documentation," AMIA Annu. Symp. ProceedingsAMIA Symp., vol. 2017, pp. 1169–1178, 2017.	USA	Structured and Unstructured Data	Progress notes/Primary care notes	None	None	Living Condition; Empoyment Status; Physical Activity; Substance Use/Abuse; Geographic Location; Marital Status	Neighborhood and Built Environment; Economic Stability; Health and Health Care; Social and Community Context	Individual	Contributed	188,120	Not specified	* NLP (information extraction, biomedicus, Stanford Probabilistic Context-Free Grammars (PCFGs) parser)<br>* Statistical methods (descriptive statistics)
S. Keyhani et al., "Using natural language processing to extract social determinants of health and improve 30-day readmission models," J. Gen. Intern. Med., vol. 32 (2 Supplement 1), p. S370, Apr. 2017.	USA	Structured and Unstructured Data	Progress notes/Primary care notes/Admission notes/Discharge summaries	risk of 30 day readmission	Admission/Readmission	Substance Use/Abuse; Housing Status; Erratic Healthcare; Living Condition; Social Support	Health and Health Care; Neighborhood and Built Environment; Social and Community Context	Individual	Contributed	8,471	Not specified	* NLP (information extraction, Moonstone)<br>* Statistical methods (WorkBench, precision, recall, accuracy, f-measure)
M. Jamei, A. Nisnevich, E. Wetchler, S. Sudat, and E. Liu, "Predicting all-cause risk of 30-day hospital readmission using artificial neural networks," PLoS ONE Electron. Resour., vol. 12, no. 7, p. e0181173, 2017, doi: https://dx.doi.org/10.1371/journal.pone.0181173.	USA	Structured Data	NA	Hospital readmission risk	Admission/Readmission	Substance Use/Abuse	Health and Health Care	Individual	Contributed	335815	Not specified	* predictive models (neural network)
B. M. Hollister, N. A. Restrepo, E. Farber-Eger, D. C. Crawford, M. C. Aldrich, and A. Non, "Development and Performance of Text-Mining Algorithms to Extract Socioeconomic Status from De-Identified Electronic Health Records," Pac. Symp. Biocomput., vol. 22, pp. 230–241, 2017, doi: https://dx.doi.org/10.1142/9789813207813_0023.	USA	Unstructured Data	Progress notes	None	None	Socioeconomic Factors; Education; Employment Status; Insurance; Housing Status	Economic Stability; Education; Health and Health Care; Neighborhood and Built Environment	Individual	Contributed	9,977	Not specified	* NLP (information extraction, seven custom NLP algorithms using 860 SES terms)<br>* Statistical methods (descriptive statistics)
J. L. Greenwald, P. R. Cronin, V. Carballo, G. Danaei, and G. Choy, "A Novel Model for Predicting Rehospitalization Risk Incorporating Physical Function, Cognitive Status, and Psychosocial Support Using Natural Language Processing," Med. Care, vol. 55, no. 3, pp. 261–266, 2017, doi: https://dx.doi.org/10.1097/MLR.0000000000000651.	USA	Unstructured Data	Progress notes/Admission notes/Discharge summaries	Hospital readmission risk	Admission/Readmission	Physical Activity; Psychosocial Factors	Health and Health Care; Social and Community Context	Individual and Neighborhood	Contributed	30,000	3	* NLP (information extraction using NLP library of terms)<br>* Predictive modeling (logistic regression, multivariable logistic regression)<br>* Statistical methods (bivariate analysis, 2-sample tests, sensitivity analysis)

Raw File: Social and behavioral determinants of health in the era of artificial intelligence with electronic health records: A scoping review

A. Davoudi, T. Ozrazgat-Baslanti, A. Ebadi, A. C. Bursian, A. Bihorac, and P. Rashidi, "Delirium Prediction using Machine Learning Models on Predictive Electronic Health Records Data," in Proceedings - 2017 IEEE 17th International Conference on Bioinformatics and Bioengineering, BIBE 2017, 2017, vol. 2018-January, pp. 568–573. doi: 10.1109/bibe.2017.00014.	USA	Structured Data	NA	delirium	Mental, Behavioral and Neurodevelopmental disorders	Substance Use/Abuse; Geographic Location; Insurance	Health and Health Care; Neighborhood and Built Environment	Individual and Neighborhood	Contributed	51,457	Not specified	* Predictive modeling (Naive Bayes (NB), generalized additive model (GAM), logistic regression (LR), support vector machine (SVM), random forests (RF), extreme gradient boosting (XGB), and neural networks (NN))<br>* Statistical methods (descriptive statistics, accuracy, AUC, sensitivity, specificity)
L. J. Anzaldi, A. Davison, C. M. Boyd, B. Leff, and H. Kharrazi, "Comparing clinician descriptions of frailty and geriatric syndromes using electronic health records: a retrospective cohort study," BMC Geriatr., vol. 17, no. 1, p. 248, 25 2017, doi: https://dx.doi.org/10.1186/s12877-017-0645-7.	USA	Unstructured Data	Progress notes/Discharge summaries/Emergency department notes	geriatric syndrome	Geriatric Syndrome	Social Support; Physical Activity	Health and Health Care; Social and Community Context	Individual	Contributed	18,341	Not specified	* NLP (pattern-based NLP algorithm, regular expressions)<br>* Statistical methods (data stratification, descriptive statistics, linear regression, Pearson correlation)
M. Afzal, M. Hussain, W. A. Khan, T. Ali, A. Jamshed, and S. Lee, "Smart Extraction and Analysis System for Clinical Research," Telemed. J. E Health, vol. 23, no. 5, pp. 404–420, 2017, doi: https://dx.doi.org/10.1089/tmj.2016.0157.	Korea, pakistan	Unstructured Data	Progress notes/Admission notes/Discharge summaries/Operative notes	cancer (but it is a case study to prove their system)	Neoplasms	Lifestyle; Substance Use/Abuse	Health and Health Care	Individual	Minimal contribution	3,811	Not specified	* NLP (tokenization, text filtering, text removal, token normalization, lexicon development)<br>* Statistical methods (descriptive statistics)
L. Wang, J. Lakin, C. Riley, Z. Korach, L. N. Frain, and L. Zhou, "Disease Trajectories and End-of-Life Care for Dementias: Latent Topic Modeling and Trend Analysis Using Clinical Notes," AMIA Annu. Symp. ProceedingsAMIA Symp., vol. 2018, pp. 1056–1065, 2018.	USA	Unstructured Data	Progress notes/Discharge summaries/Emergency department notes	dementia	Mental, Behavioral and Neurodevelopmental disorders	Physical Activity; Education; Marital Status	Health and Health Care; Education; Social and Community Context	Individual	Contributed	7,875	Not specified	* NLP (information extraction, LDA topic modeling)<br>* Predictive modeling (LDA topic modeling)<br>* Statistical methods (descriptive statistics)
Q. Chen et al., "Predicting suicide attempt or suicide death following a visit to psychiatric specialty care: A machine learning study using Swedish national registry data," PLoS Med. Public Libr. Sci., vol. 17, no. 11, p. e1003416, Nov. 2020, doi: https://dx.doi.org/10.1371/journal.pmed.1003416.	USA	Structured Data	NA	non-fatal suicide attempt	Mental, Behavioral and Neurodevelopmental disorders	Socioeconomic Factors	Economic Stability	Neighborhood	Contributed	37310	Not specified	* Predictive modeling (random forests (RF))<br>* Statistical methods (AUC, precision, recall, Brier score)<br>* Other (BioVU Synthetic Derivative (SD))
D. A. Thompson, D. M. Courtney, S. Malik, M. Schmidt, and V. Weston, "Use of natural language processing to identify 414 different chief complaints in adult emergency department patients," Acad. Emerg. Med., vol. 25 (Supplement 1), p. S193, May 2018, doi: http://dx.doi.org/10.1111/acem.13424.	USA	Structured and Unstructured Data	Progress notes/Emergency department notes	Multiple Diseases	Miscellaneous	Substance Use/Abuse	Health and Health Care	Individual	Contributed	86,948	1	* NLP (data standardization, NLP engine (Health Navigator LLC))<br>* Statistical methods (descriptive statistics)
J. Shi, X. Fan, J. Wu, J. Chen, and W. Chen, "DeepDiagnosis: DNN-Based Diagnosis Prediction from Pediatric Big Healthcare Data," in Proceedings - 2018 6th International Conference on Advanced Cloud and Big Data, CBD 2018, 2018, pp. 287–292. doi: 10.1109/cbd.2018.00058.	China	Structured and Unstructured Data	Progress notes	Pediatric diseases	Miscellaneous	Environmental Factors	Neighborhood and Built Environment	Neighborhood	Contributed	4000000	1	* NLP (vectorization, Word2Vec)<br>* Predictive modeling (CNN, RNN, LSTM, BiLSTM, BiRNN)<br>* Statistical methods (descriptive statistics, precision)
F. Rahimian et al., "Predicting the risk of emergency admission with machine learning: Development and validation using linked electronic health records," PLoS Med. Public Libr. Sci., vol. 15, no. 11, p. e1002695, 2018, doi: https://dx.doi.org/10.1371/journal.pmed.1002695	UK	Structured Data	NA	first emergency admission prediction	Admission/Readmission	Lifestyle Factors; Socioeconomic Status; Substance Use/Abuse	Health and Health Care; Economic Stability	Individual	Contributed	4,600,000	389	* Predictive modeling (Cox proportional hazards)
A. S. Navathe et al., "Hospital Readmission and Social Risk Factors Identified from Physician Notes," Health Serv. Res., vol. 53, no. 2, pp. 1110–1136, 2018, doi: https://dx.doi.org/10.1111/1475-6773.12670.	USA	Structured and Unstructured Data; Claims Data	Discharge summaries/Progress notes / Admission notes	Cardiovascular disease	Diseases of the circulatory system	Substance Use/Abuse; Housing Instability; Social Support	Economic Stability; Health and Health Care; Social and Community Context	Individual	Contributed	49,319	1	* NLP (MTERMS)<br>* Statistical methods (Chi-square, multivariable logistic regression model, bivariate logistic regression, sensitivity analysis)
H. Kharrazi et al., "The Value of Unstructured Electronic Health Record Data in Geriatric Syndrome Case Identification," J. Am. Geriatr. Soc., vol. 66, no. 8, pp. 1499–1507, 2018, doi: https://dx.doi.org/10.1111/jgs.15411.	USA	Structured and Unstructured Data; Claims Data	Progress notes/Discharge summaries/Emergency Department Notes	Geriatric syndrome	Geriatric Syndrome	Social Support	Social and Community Context	Individual	Contributed	18,341	4+	* NLP (information extraction)<br>* Statistical methods (data stratification, descriptive statistics)
A. Hassoon et al., "Increasing Physical Activity Amongst Overweight and Obese Cancer Survivors Using an Alexa-Based Intelligent Agent for Patient Coaching: Protocol for the Physical Activity by Technology Help (PATH) Trial," JMIR Res. Protoc., vol. 7, no. 2, pp. e27–e27, Feb. 2018, doi: 10.2196/resprot.9096.	USA	Structured Data	NA	Cancer, diseases that are common among cancer survivors, diseases associated with being obese or overwight	Neoplasms	Education,; Health Literacy; Lifestyle; Diet	Education; Health and Health Care	Individual	Contributed	42	1	* NLP (conversational bot, intelligent agent, chatbot)<br>* Predictive modeling (linear regression)<br>* Statistical methods (linear regression, descriptive statistics)
Z. M. Grinspan, A. D. Patel, B. Hafeez, E. L. Abramson, and L. M. Kern, "Predicting frequent emergency department use among children with epilepsy: A retrospective cohort study using electronic health data from 2 centers," Epilepsia, vol. 59, no. 1, pp. 155–169, 2018, doi: https://dx.doi.org/10.1111/epi.13948.	USA	Structured Data	NA	Epilepsy	Mental, Behavioral and Neurodevelopmental disorders	Health Access; Health Literacy; Insurance	Education; Health and Health Care	Individual and Neighborhood	Contributed	3,516	2	* Predictive modeling (3-variable model, random forests (RF))<br>* Statistical methods (Bivariate analysis)
D. J. Feller, J. Zucker, M. T. Yin, P. Gordon, and N. Elhadad, "Using Clinical Notes and Natural Language Processing for Automated HIV Risk Assessment," J. Acquir. Immune Defic. Syndr. JAIDS, vol. 77, no. 2, pp. 160–166, 01 2018, doi: https://dx.doi.org/10.1097/QAI.0000000000001580.	USA	Structured and Unstructured Data	Progress notes/Primary care notes/Admission notes/Discharge summaries	HIV	Certain infectious and parasitic diseases	Social and behavioral determinants of health	Social and Community Context	Individual and Neighborhood	Contributed	181	Not specified	* NLP (term frequency-inverse document frequency (TF-IDF), LDA topic modeling)<br>* Predictive modeling (random forests (RF), LDA topic modeling))<br>* Statistical methods (descriptive statistics, ROC, F1-score)
D. J. Feller et al., "Towards the Inference of Social and Behavioral Determinants of Sexual Health: Development of a Gold-Standard Corpus with Semi-Supervised Learning," AMIA Annu. Symp. ProceedingsAMIA Symp., vol. 2018, pp. 422–429, 2018.	USA	Structured and Unstructured Data	Progress notes/Primary care notes/Admission notes/Discharge summaries	No specific disease	None	Social and behavioral determinants of health; Substance Use/Abuse; Housing Status	Social and Community Context; Health and Health Care; Neighborhood and Built Environment	Individual and Neighborhood	Contributed	343,322	Not specified	*NLP (word embedding)-Semi-Supervised Learning, Support Vector Machine classifier (SVM)
J. Erickson, K. Abbott, and L. Susienka, "Automatic address validation and health record review to identify homeless Social Security disability applicants," J. Biomed. Inform., vol. 82, pp. 41–46, 2018, doi: https://dx.doi.org/10.1016/j.jbi.2018.04.012.	USA	Structured and Unstructured Data; Claims Data	Clinical notes	No disease specified	None	Housing Status; Housing Instability; Education; Substance Use/Abuse; Employment Status	Neighborhood and Built Environment; Economic Stability; Education; Health and Health Care	Individual and Neighborhood	Contributed	4,628	Not specified	* NLP (information extraction, data annotation, MetaMap, UMLS)<br>* Predictive modeling (random forests (RF))<br>* Statistical methods (descriptive statistics, sensitivity, specificity, TP, FP, TN, FN, PPV, NPV)
A. Dagliati et al., "A dashboard-based system for supporting diabetes care," J. Am. Med. Inform. Assoc., vol. 25, no. 5, pp. 538–547, 01 2018, doi: https://dx.doi.org/10.1093/jamia/ocx159.	Italy, UK, Spain	Unstructured Data	Progress notes	Diabetes	Endocrine, nutritional and metabolic diseases	Lifestyle	Health and Health Care	Individual	Contributed	700	Not specified	* Predictive modeling (longitudinal data analytics, JTSA, CFM, pattern detection algorithms, risk-prediction models)
K. M. Corey et al., "Development and validation of machine learning models to identify high-risk surgical patients using automatically curated electronic health record data (Pythia): A retrospective, single-site study," PLoS Med. Public Libr. Sci., vol. 15, no. 11, p. e1002701, 2018, doi: https://dx.doi.org/10.1371/journal.pmed.1002701.	USA	Structured Data	NA	Not specified, patients with the disease that have to get a surgery	None	Substance Use/Abuse	Health and Health Care	Individual	Contributed	163,599	Not specified	* Predictive modeling (lasso, random forests (RF), extreme gradient boosted decision trees)<br>* Statistical methods (AUC)
N. A. Bhavsar, A. Gao, M. Phelan, N. J. Pagidipati, and B. A. Goldstein, "Value of Neighborhood Socioeconomic Status in Predicting Risk of Outcomes in Studies That Use Electronic Health Record Data," JAMA Netw. Open, vol. 1, no. 5, p. e182716, 07 2018, doi: https://dx.doi.org/10.1001/jamanetworkopen.2018.2716.	USA	Structured Data	NA	No specific disease	None	Social Environment; Socioeconomic Status; Environmental Factors	Social and Community Context; Economic Stability; Neighborhood and Built Environment	Neighborhood	Contributed	90,097	2	* Predictive modeling (random survival forest (RSF))<br>* Statistical methods (descriptive statistics, R-squared, AUC, C-statistic)

C. A. Bejan et al., “Mining 100 million notes to find homelessness and adverse childhood experiences: 2 case studies of rare and severe social determinants of health in electronic health records,” J. Am. Med. Inform. Assoc., vol. 25, no. 1, pp. 61–71, 01 2018, doi: https://dx.doi.org/10.1093/jamia/ocx059.	USA	Unstructured Data	Clinical notes	No specific disease	None	Social and behavioral determinants of health; Housing Status	Social and Community Context; Neighborhood and Built Environment	Individual and Neighborhood	Contributed	3544	1	* NLP (information retrieval, word2vec)<br>* Statistical methods (descriptive statistics, precision, recall, AUPRC)
V. J. Zhu, L. A. Lenert, B. E. Bunnell, J. S. Obeid, M. Jefferson, and C. H. Halbert, “Automatically identifying social isolation from clinical narratives for patients with prostate Cancer,” BMC Med. Inform. Decis. Mak., vol. 19, no. 1, p. 43, 14 2019, doi: https://dx.doi.org/10.1186/s12911-019-0795-y.	USA	Unstructured Data	NA	Prostate cancer	Neoplasms	Social Isolation; Social Support; Social Environment	Social and Community Context	Individual and Neighborhood	Contributed	3,138	1	* NLP (information retrieval, UMLS metathesaurus, (I2E))<br>* Statistical methods (descriptive statistics, precision, recall, F1-score)
X. Zhou, Y. Wang, S. Sohn, T. M. Therneau, H. Liu, and D. S. Knopman, “Automatic extraction and assessment of lifestyle exposures for Alzheimer’s disease using natural language processing,” Int. J. Med. Inf., vol. 130, p. 103943, 2019, doi: https://dx.doi.org/10.1016/j.ijmedinf.2019.08.003.	USA	Unstructured Data	Primary care notes	Alzheimer's disease, dementia, AD dementia	Mental, Behavioral and Neurodevelopmental disorders	Lifestyle; Diet; Substance Use/Abuse	Health and Health Care	Individual	Contributed	260	1	* NLP (information retreival, MetaMap, UMLS, dictionary construction, NLTK, annotation, gold standard dataset development)<br>* Statistical methods (chi-square, Student's t-test, generalized linear models, confidence intervals)
A. Shaham, G. Chodick, V. Shalev, and D. Yamin, “Personal and social patterns predict influenza vaccination decision,” BMC Public Health, vol. 20, no. 1, p. 222, Feb. 2020, doi: https://dx.doi. org/10.1186/s12889-020-8327-3.	Israel	Structured Data	NA	Influenza	Diseases of the respiratory system	Environmental Factors; Social and behavioral determinants of health	Neighborhood and Built Environment; Social and Community Context	Individual	Contributed	250,000	1	* Predictive modeling (logistic regression, Naive Bayes, XGBoost Random Forest, Light GBM Random Forest, Artificial Neural Network)<br>* Statistical methods (descriptive statistics, AUC, ROC, precision, recall, F1-score)
Q. Xue, X. Wang, S. Meehan, J. Kuang, J. A. Gao, and M. C. Chuah, “Recurrent Neural Networks Based Obesity Status Prediction Using Activity Data,” in Proceedings - 17th IEEE International Conference on Machine Learning and Applications, ICMLA 2018, 2019, pp. 865–870. doi: 10.1109/icmla. 2018.00139.	USA	Structured Data	NA	Diseases related to obesity, hypertension, stsroke, and type 2 diabetes	Endocrine, nutritional and metabolic diseases; Diseases of the circulatory system	Housing Instability; Education; Employment Status; Marital Status	Economic Stability; Education; Social and Community Context	Individual	Contributed	275	Not specified	* NLP (data normalization)<br>* Predictive modeling (logistic regression (LR) and random forests (RF), Long Short-term Memory (LSTM), recurrent neural network (RNN))<br>* Statistical methods (accuracy)
E. A. Wang et al., “Measuring Exposure to Incarceration Using the Electronic Health Record,” Med. Care, vol. 57 Suppl 6 Suppl 2, pp. S157–S163, 2019, doi: https://dx.doi.org/10.1097/MLR. 0000000000001049.	USA	Unstructured Data	Progress notes	human immunodeficiency virus - HIV disease	Certain infectious and parasitic diseases	Incarceration; Racial Disparities; Socioeconomic Status; Social Discrimination	Social and Community Context; Economic Stability	Individual and Neighborhood	Contributed	57,765	1	* NLP (information extraction, data annotation, Yale cTAKES extension (YTEX), support vector machine (SVM))<br>* Predictive modeling (support vector machine (SVM) based classification task)<br>* Statistical methods (F1-score, precision, Matthews correlation coefficient)
J. R. Vest and O. Ben-Assuli, “Prediction of emergency department revisits using area-level social determinants of health measures and health information exchange information,” Int. J. Med. Inf., vol. 129, pp. 205–210, 2019, doi: https://dx.doi.org/10. 1016/j.ijmedinf.2019.06.013.	USA, Israel	Structured Data	NA	Not a single focus but on general chronic diseases	Chronic Diseases	Socioeconomic Status; Social Behavior; Socio-environmental Neighbourhood; Health Access; Social Circumstances	Economic Stability; Social and Community Context; Neighborhood and Built Environment; Health and Health Care	Individual and Neighborhood	Contributed	279,611	Adult Emeregency Department paitents	* Predictive modeling (two-class boosted decision trees)<br>* Statistical methods (AUC, ROC, accuracy, precision, recall, F1-score)
B. Olatosi, J. Zhang, S. Weissman, J. Hu, M. R. Haider, and X. Li, “Using big data analytics to improve HIV medical care utilisation in South Carolina: A study protocol,” BMJ Open, vol. 9, no. 7, p. e027688, 19 2019, doi: https://dx.doi.org/10.1136/bmjopen-2018-027688.	USA	Structured Data	NA	HIV	Certain infectious and parasitic diseases	Social Support; Social Behavior	Social and Community Context	Neighborhood	Contributed	18,998	Not specified	* Predictive modeling (logistic regression, naive Bayes classifier, support vector machine and random forest)
J. P. Lalor, B. Woolf, and H. Yu, “Improving Electronic Health Record Note Comprehension With NoteAid: Randomized Trial of Electronic Health Record Note Comprehension Interventions With Crowdsourced Workers,” J. Med. Internet Res., vol. 21, no. 1, p. e10793, 16 2019, doi: https://dx.doi.org/10.2196/10793.	USA	Structured and Unstructured Data	Clinical notes	no specific disease studied (but the dataset included information about: heart failure, hypertension, diabetes, chronic obstructive pulmonary disease (COPD), liver failure, and cancer)	Multiple Diseases	Health Literacy; Employment Status; Education	Economic Stability; Education	Individual and Neighborhood	Contributed	97	Not specified	* Predictive modeling (linear regression)
K.-M. Kuo, P. C. Talley, M. Kuzuya, and C.-H. Huang, “Development of a clinical support system for identifying social frailty,” Int. J. Med. Inf., vol. 132, p. 103979, 2019, doi: https: //dx.doi.org/10.1016/j.ijmedinf.2019.103979.	China (Taiwan), Japan	Structured Data	NA	clinical geriatric syndrome	Geriatric Syndrome	Lifestyle; Physical Activity; Diet; Socioeconomic Factors	Economic Stability; Health and Health Care	Neighborhood	Contributed	595	Not specified	* Predictive modeling (bagged classification and regression trees (CART), model average neural network, random forests (RF), eXtreme gradient boosting, and stochastic gradient boosting)<br>* Statistical methods (descriptive statistics, AUC, Kappa, sensitivity, specificity)
I. K. Kirk et al., “Linking glycemic dysregulation in diabetes to symptoms, comorbidities, and genetics through EHR data mining,” eLife, vol. 8, no. 12, p. 10, 10 2019, doi: https://dx.doi. org/10.7554/eLife.44941.	Denmark	Unstructured Data	Primary care notes/ Progress notes	diabetes	Endocrine, nutritional and metabolic diseases	Lifestyle	Health and Health Care	Neighborhood	Contributed	14,017	Not specified	* NLP (text mining, unsupervised Markov clustering)<br>* Predictive modeling (unsupervised Markov clustering)
A. Ferri et al., “Towards the Design of a Machine Learning-based Consumer Healthcare Platform powered by Electronic Health Records and measurement of Lifestyle through Smartphone Data,” in 2019 IEEE 23rd International Symposium on Consumer Technologies, ISCT 2019, 2019, pp. 37–40. doi: 10.1109/isce. 2019.8901034.	Italy	Structured Data	NA	Nothing mentioned (only BA and CA - biological age and chronological age)	None	Lifestyle; Social Behaviour; Socioeconomic factors	Health and Health Care; Social and Community Context; Economic Stability	Individual and Neighborhood	Minimal contribution	484	Not specified	* Predictive modeling (supervised regression models)
D. A. DuBay et al., “Development and future deployment of a 5 years allograft survival model for kidney transplantation,” Nephrology, vol. 24, no. 8, pp. 855–862, Aug. 2019, doi: https: //dx.doi.org/10.1111/nep.13488.	USA	Structured and Unstructured Data	Progress notes	allograft survival		Education; Income	Education; Economic Stability	Individual and Neighborhood	Contributed	1439	Clinical Website	* Predictive modeling (big data approach, multi-variable cox regression model)
D. Dorr, C. A. Bejan, C. Pizzimenti, S. Singh, M. Storer, and A. Quinones, “Identifying Patients with Significant Problems Related to Social Determinants of Health with Natural Language Processing,” Stud. Health Technol. Inform., vol. 264, pp. 1456–1457, Aug. 2019, doi: https://dx.doi.org/10.3233/SHTI190482.	USA	Structured and Unstructured Data	Progress notes/Primary care notes	None	None	Psychosocial Factors; Social Isolation; Financial Insecurity; Housing Instability	Social and Community Context; Economic Stability	Individual and Neighborhood	Contributed	358,000	Not specified	* NLP (information retrieval, tokenization, lexical association, term frequency-inverse document frequency (TF-IDF))
T. Chen, M. Dredze, J. P. Weiner, and H. Kharrazi, “Identifying vulnerable older adult populations by contextualizing geriatric syndrome information in clinical notes of electronic health records,” J. Am. Med. Inform. Assoc., vol. 26, no. 8–9, pp. 787–795, 01 2019, doi: https://dx.doi.org/10.1093/jamia/ocz093.	USA	Unstructured Data	Progress notes	geriatric syndromes	Geriatric Syndrome	Social Support	Social and Community Context	Individual	Contributed	185	Not specified	* NLP (statistical NLP model, deep learning system for sentence classification that incorporates contextual information from surrounding sentences, the entire document, and structured diagnostic codes)<br>* Statistical methods (F1-score)
T. Byrne, A. E. Montgomery, and J. D. Fargo, “Predictive modeling of housing instability and homelessness in the Veterans Health Administration,” Health Serv. Res., vol. 54, no. 1, pp. 75–85, 2019, doi: https://dx.doi.org/10.1111/1475-6773.13050.	USA	Structured Data	NA	None	None	Housing Instability; Housing Status	Economic Stability; Neighborhood and Built Environment	Individual	Contributed	5800000	Not specified	* Predictive modeling (logistic regression and random forests (RF))
B. T. Bucher, J. Shi, R. J. Pettit, J. Ferraro, W. W. Chapman, and A. Gundlapalli, “Determination of Marital Status of Patients from Structured and Unstructured Electronic Healthcare Data,” AMIA Annu. Symp. ProceedingsAMIA Symp., vol. 2019, pp. 267–274, 2019.	USA	Structured and Unstructured Data	Progress notes	None	None	Marital Status	Social and Community Context	Individual and Neighborhood		4716	Not specified	* NLP (EasyCIE)<br>* Predictive modeling (random forests (RF))

S. A. Berkowitz, S. Basu, A. Venkataramani, G. Reznor, E. W. Fleegler, and S. J. Atlas, "Association between access to social service resources and cardiometabolic risk factors: A machine learning and multilevel modeling analysis," BMJ Open, vol. 9, no. 3, 2019, doi: http://dx.doi.org/10.1136/bmjopen-2018-025281.	USA	Structured Data	NA	None	None	Health Access; Housing Status; Diet; Employment Status; Violence; Substance Use/Abuse; Education	Health and Health Care; Neighborhood and Built Environment; Economic Stability; Education	Individual and Neighborhood	Contributed	123,355	Not specified	* Predictive modeling (multilevel linear mixed models)
M. Afshar et al., "Subtypes in patients with opioid misuse: A prognostic enrichment strategy using electronic health record data in hospitalized patients," PLoS ONE Electron. Resour., vol. 14, no. 7, p. e0219717, 2019, doi: https://dx.doi.org/10.1371/journal.pone.0219717.	USA	Structured and Unstructured Data	Discharge summaries/Progress notes / Admission notes	Opioid misuse	Mental, Behavioral and Neurodevelopmental disorders	Socioeconomic Status; Insurance; Education; Employment Status; Housing Status	Economic Stability; Education; Health and Health Care; Neighborhood and Built Environment	Individual and Neighborhood	Contributed	6,224	Not specified	* NLP (Apache clinical Text Analysis Knowledge Extraction System (cTAKES), Latent Dirichlet Allocation (LDA), topic modeling)<br>* Statistical methods (descriptive statistics)
M. Conway et al., "Moonstone: a novel natural language processing system for inferring social risk from clinical narratives," J. Biomed. Semant., vol. 10, no. 1, p. 6, 11 2019, doi: https://dx.doi.org/10.1186/s13326-019-0198-0.	USA	Unstructured Data	NA	congestive heart failure, acute myocardial infarction, pneumonia, and stroke	None	housing situation, living alone, and social support		Individual	Contributed	500	1	NLP system (Moonstone)
L. Zheng et al., "Development of an early-warning system for high-risk patients for suicide attempt using deep learning and electronic health records," Transl Psychiatry Psychiatry, vol. 10, no. 1, p. 72, 20 2020, doi: https://dx.doi.org/10.1038/s41398-020-0684-2.	USA, China	Structured Data	NA	Suicide	Mental, Behavioral and Neurodevelopmental disorders	Socioeconomic Status; Education level; Employment Status; Income; Socioeconomic Factors	Economic Stability; Education	Neighborhood	Contributed	118,252	3	* Predictive modeling (deep learning model, logistic regression, XGBoost)<br>* Statistical methods (AUC, descriptive statistics)
Y. Zhang-James, Q. Chen, R. Kuja-Halkola, P. Lichtenstein, H. Larsson, and S. V. Faraone, "Machine-Learning prediction of comorbid substance use disorders in ADHD youth using Swedish registry data," J. Child Psychol. Psychiatry, vol. 01, p. 01, Apr. 2020, doi: https://dx.doi.org/10.1111/jcpp.13226.	USA, Sweden	Structured Data	NA	attention-deficit/hyperactivity disorder (ADHD) and substance use disorders (SUDs)	Mental, Behavioral and Neurodevelopmental disorders	Education; Socioeconomic Status; Geographic Location	Education; Economic Stability; Neighborhood and Built Environment	Individual	Contributed	19,184	Not specified	* Predictive modeling (random forests (RF), longitudinal recurrent neural network (RNN), longitudinal recurrent neural network (RNN) model with the Long Short-Term Memory (LSTM))
L. Williamson et al., "Finding Undiagnosed Patients with Familial Hypercholesterolemia in Primary Care Usingelectronic Health Records," J. Am. Coll. Cardiol., vol. 75 (11), p. 3502, Mar. 2020, doi: http://dx.doi.org/10.1016/S0735-1097%2820%2934129-2.	USA	Unstructured Data	Radiology reports/Pathology reports/Transcription reports	Familial hypercholesterolemia	Endocrine, nutritional and metabolic diseases	Diet	Health and Health Care	Individual	Contributed	12253	Not specified	* NLP (internal text processing workflow)
L. Wang et al., "Applying Machine Learning Models to Predict Medication Nonadherence in Crohn's Disease Maintenance Therapy," Patient Prefer. Adherence, vol. 14, pp. 917–926, 2020, doi: https://dx.doi.org/10.2147/PPA.S253732.	China	Structured and Unstructured Data	Progress notes	Crohn's Disease	Diseases of the digestive system	Socioeconomics Factors	Economic Stability	Individual	Contributed	446	1	* Predictive modeling (back-propagation neural network (BPNN) , support vector machine (SVM), logistic regression (LR))
C. Volij and S. Esteban, "Development of a Systematic Text Annotation Standard to Extract Social Support Information form Electronic Medical Records," Stud. Health Technol. Inform., vol. 270, pp. 1261–1262, Jun. 2020, doi: https://dx.doi.org/10.3233/SHTI200392.	Argentina	Unstructured Data	Clinical notes	No disease specified	None	Social Support	Social and Community Context	Individual	Contributed	2000	Not specified	* NLP (annotation standard development)
A. Tragomalou et al., "Novel e-health applications for the management of cardiometabolic risk factors in children and adolescents in greece," Nutrients, vol. 12, no. 5, 2020, doi: http://dx.doi.org/10.3390/nu12051380.	Greece	Structured Data	NA	Obesity	Endocrine, nutritional and metabolic diseases	Diet	Health and Health Care	Individual	Contributed	800	Not specified	* Predictive modeling (intelligent multi-level information systems, specialized artificial intelligence algorithms)
K. Shoenbill, Y. Song, L. Gress, H. Johnson, M. Smith, and E. A. Mendonca, "Natural language processing of lifestyle modification documentation," Health Informatics J., vol. 26, no. 1, pp. 388–405, 2020, doi: https://dx.doi.org/10.1177/1460458218824742.	USA	Unstructured Data	Primary care notes/ Progress notes	Not specified	None	Lifestyle	Health and Health Care	Individual	Contributed	14,860	Not specified	* NLP (lexicon development, information retrival, CUI mapping, data filtering, SNOMEDCT, UMLS, open source NLP tool)<br>* Statistical methods (recall, precision, NLP retrieval of LM assessment)
K. Shoenbill, Y. Song, M. Craven, H. Johnson, M. Smith, and E. A. Mendonca, "Identifying patterns and predictors of lifestyle modification in electronic health record documentation using statistical and machine learning methods," Prev. Med., vol. 136, p. 106061, 2020, doi: https://dx.doi.org/10.1016/j.ypmed.2020.106061.	USA	Structured Data	NA	hypertension	Diseases of the circulatory system	Lifestyle	Health and Health Care	Individual	Contributed	14,360	Not specified	* Predictive modeling (random forests (RF), logistic regression)
D. Yamin, A. Shaham, G. Chodick, and V. Shalev, "Personal and social patterns predict influenza vaccination decision," Isr. J. Health Policy Res. Conf. 7th Int. Jerus. Conf. Health Policy Isr., vol. 8, no. Supplement 1, 2019, doi: http://dx.doi.org/10.1186/s13584-019-0336-2.	Israel	Structured Data	NA	Seasonal influenza	Diseases of the respiratory system	Socioeconomic Status	Economic Stability	Individual	Contributed	250,000	Not specified	* Predictive modeling (logistic regression, Naive Bayes, XGBoost Random Forest, Light GBM Random Forest, Artificial Neural Network)<br>* Statistical methods (descriptive statistics, AUC, ROC, precision, recall, F1-score)
A. Seveso, V. Bozzetti, P. Tagliabue, M. L. Ventura, and F. Cabitza, "Developing a machine learning model for predicting postnatal growth in very low birth weight infants," in HEALTHINF 2020 - 13th International Conference on Health Informatics, Proceedings; Part of 13th International Joint Conference on Biomedical Engineering Systems and Technologies, BIOSTEC 2020, 2020, pp. 490–497.	Italy	Structured Data	NA	Postnatal Growth		Socioeconomic Status	Economic Stability	Individual	Contributed	964	Not specified	* Predictive modeling (regression model)<br>* Statistical methods (descriptive statistics, accuracy, AUC, MCC)
M. Senior et al., "Identifying Predictors of Suicide in Severe Mental Illness: A Feasibility Study of a Clinical Prediction Rule (Oxford Mental Illness and Suicide Tool or OxMIS)," Front. Psychiatry Front. Res. Found., vol. 11, p. 268, 2020, doi: https://dx.doi.org/10.3389/fpsyt.2020.00268.	UK	Structured and Unstructured Data	Primary care notes/ Progress notes	schizophrenia-spectrum disorders or bipolar disorder	Mental, Behavioral and Neurodevelopmental disorders	Education	Education	Individual	Contributed	54	Not specified	* NLP (vectorization, named entity recognition, gold standard dataset development)
A. H. S. Harris, A. C. Kuo, T. R. Bowe, L. Manfredi, N. F. Lalani, and N. J. Giori, "Can Machine Learning Methods Produce Accurate and Easy-to-Use Preoperative Prediction Models of One-Year Improvements in Pain and Functioning After Knee Arthroplasty?," J. Arthroplasty, vol. 20, p. 20, Jul. 2020, doi: https://dx.doi.org/10.1016/j.arth.2020.07.026.	USA	Structured Data	NA	Total knee arthroplasty (TKA)	Injury, poisoning and certain other consequences of external causes	Marital Status; Education; Employment Status	Economic Stability; Education; Social and Community Context	Individual	Contributed	637	3	* Predictive modeling (logistic regression, LASSO regression, GBM, quadratic discriminant analysis (QDA))<br>* Statistical methods (C-statistic, Brier score)
F. Ge, J. Jiang, Y. Wang, C. Yuan, and W. Zhang, "Identifying suicidal ideation among chinese patients with major depressive disorder: Evidence from a real-world hospital-based study in China," Neuropsychiatr. Dis. Treat., vol. 16, pp. 665–672, 2020, doi: http://dx.doi.org/10.2147/NDT.S238286.	China	Structured Data	NA	Major depressive disorder	Mental, Behavioral and Neurodevelopmental disorders	Maritial Status; Vocational History	Economic Stability; Social and Community Context	Individual	Contributed	1,916	1	* Predictive modeling (machine learning algorithm, neural network<br>* Statistical methods (descriptive statistics)
C. Dalton-Locke, J. H. Thygesen, N. Werbeloff, D. Osborn, and H. Killaspy, "Using de-identified electronic health records to research mental health supported housing services: A feasibility study," PLoS ONE Electron. Resour., vol. 15, no. 8, p. e0237664, 2020, doi: https://dx.doi.org/10.1371/journal.pone.0237664.	UK	Unstructured Data	Progress notes	Mental health	Mental, Behavioral and Neurodevelopmental disorders	Housing Insecurity; Housing Status	Economic Stability; Neighborhood and Built Environment	Individual	Contributed	2,140	Not specified	* NLP (free text search)
W. Cui, D. Robins, and J. Finkelstein, "Unsupervised Machine Learning for the Discovery of Latent Clusters in COVID-19 Patients Using Electronic Health Records," Stud. Health Technol. Inform., vol. 272, pp. 1–4, Jun. 2020, doi: https://dx.doi.org/10.3233/SHTI200478.	USA	Structured Data	NA	COVID-19	Diseases of the respiratory system	Socioeconomic Factors	Economic Stability	Individual	Contributed	6,000	1	* Predictive modeling (big data analytics, unsupervised machine learning, Clustering, elbow method)
A. T. Bako, H. Walter-McCabe, S. N. Kasthurirathne, P. K. Halverson, and J. R. Vest, "Reasons for Social Work Referrals in an Urban Safety-Net Population: A Natural Language Processing and Market Basket Analysis Approach," J. Soc. Serv. Res., 2020, doi: 10.1080/01488376.2020.1817834.	USA	Structured and Unstructured Data	Primary Care notes	None	None	Financial Insecurity; Social Support	Economic Stability; Social and Community Context	Individual	Contributed	9,473	1	* NLP (data categorization)<br>* Statistical methods (Market based analysis (MBA))

K. Lybarger, M. Ostendorf, and M. Yetisgen, "Annotating social determinants of health using active learning, and characterizing determinants using neural event extraction," J. Biomed. Inform., vol. 113, p. 103631, Jan. 2021, doi: 10.1016/j.jbi.2020.103631.	USA	Unstructured Data	Discharge summaries/Progress notes / Admission notes/Emergency department notes	None	None	Substance Use/Abuse; Physical Activity; Employment Status; Insurance; Living Condition; Environmental Factors	Health and Health Care; Economic Stability; Neighborhood and Built Environment	Individual and Neighborhood	Contributed	3136	2	* NLP (data annotation), A neural multi-task model, Event Extractor (Nueral Networks)
S. Keyhani et al., "Using natural language processing to extract social determinants of health and improve 30-day readmission models," J. Gen. Intern. Med., vol. 32 (2 Supplement 1), p. S370, Apr. 2017.	USA	Unstructured Data	clinical notes	None	None	Homelessness	Neighborhood and Built Environment	Individual	Contributed	2229983 veterans ; 500 notes	2	* NLP (data annotation, information retrival (NLP tool: Automated Retrieval Console))
A. V. Gundlapalli et al., "Extracting Concepts Related to Homelessness from the Free Text of VA Electronic Medical Records," AMIA Annu. Symp. Proc. AMIA Symp., vol. 2014, pp. 589–598, Nov. 2014.	USA	Unstructured Data	clinical notes	None	None	Homelessness	Neighborhood and Built Environment	Individual	Contributed	Not mentioned	Veterans Information and Computing Infrastructure (VINCI) documents: 862 documents.	* NLP (V3NLP Framework, lexical analysis, PPV)
R. Suchting, C. E. Green, S. M. Glazier, and S. D. Lane, "A data science approach to predicting patient aggressive events in a psychiatric hospital," Psychiatry Res., vol. 268, pp. 217–222, Oct. 2018, doi: 10.1016/j.psychres.2018.07.004.	USA	Structured Data	clinical notes	Aggression	Mental, Behavioral and Neurodevelopmental disorders	Homelessness	Neighborhood and Built Environment	Individual	Contributed	29,841	1	* Predictive modeling (GLM, RF, GBM, DNN)
E. Brignone, J. D. Fargo, R. K. Blais, and A. V. Gundlapalli, "Applying Machine Learning to Linked Administrative and Clinical Data to Enhance the Detection of Homelessness among Vulnerable Veterans," AMIA Annu. Symp. Proc. AMIA Symp., vol. 2018, pp. 305–312, Dec. 2018.	USA	Unstructured Data	clinical notes	None	None	Homelessness	Neighborhood and Built Environment	Individual	Contributed	25,510	2 (national clinical data from VHA and the Department of Defense.)	* Predictive modeling (Random forest)
D. J. Feller, O. J. Bear Don't Walk Iv, J. Zucker, M. T. Yin, P. Gordon, and N. Elhadad, "Detecting Social and Behavioral Determinants of Health with Structured and Free-Text Clinical Data," Appl. Clin. Inform., vol. 11, no. 1, pp. 172–181, Jan. 2020, doi: 10.1055/s-0040-1702214.	USA	Structured and Unstructured Data	clinical notes	None	None	alcohol abuse, substance abuse, and housing status (homelessness)	Health and Health Care; Neighborhood and Built Environment	Individual	Contributed	1,501 patients	1(CUMU)	* Predictive modeling (TF-IDF, SVM, Random Forest, Logistic Regression, classification tree, adaboost)
D. Schillinger, R. Balyan, S. A. Crossley, D. S. McNamara, J. Y. Liu, and A. J. Karter, "Employing computational linguistics techniques to identify limited patient health literacy: Findings from the ECLIPPSE study," Health Serv. Res., vol. 23, p. 23, Sep. 2020, doi: https://dx.doi.org/10.1111/1475-6773.13560.	USA	Structured and Unstructured Data	clinical notes	Diabetes	Endocrine, nutritional and metabolic diseases	Health Literacy	Education	Individual	Contributed	6941 diabetes patients	283216 messages, KPNC portal	* NLP (lexical analysis; Lingustic indices, Tool for the Automatic Assessment of Lexical Sophistication, the Tool for the Automatic Analysis of Cohesion, the Tool for the Automatic Assessment of Syntactic Sophistication and Complexity, the SEntiment ANalysis and Cognition Engine, and Coh-Metrix.)
