# Supplementary material for: Social and Behavioral Determinants of Health in the Era of Artificial Intelligence with Electronic Health Records: A Scoping Review
Source: Health Data Sci. 2021 Aug 24;2021:9759016. doi: 10.34133/2021/9759016 (PMC10880156; doi:10.34133/2021/9759016)
Supplement: Supplementary Materials — Supplemental Document 1: literature searching strategies on Ovid, Scopus, Web of Science, ACM digital library, and IEEE Xplore. Supplementary Table 1: information extracted from the included studies. [file 9759016.f1.zip › SBDH Search Strategies.docx]

# Ovid

Database(s): Embase 1974 to 2021 April 07 , Ovid MEDLINE® and Epub Ahead of Print, In-Process, In-Data-Review & Other Non-Indexed Citations and Daily 1946 to April 07, 2021
Search Strategy:

| **#** | **Searches** | **Results** |
| --- | --- | --- |
| 1 | (EHR or EHRs or “electronic health record” or “electronic health records” or “electronic medical record” or “electronic medical records” or EMR or EMRs).ti,ab,hw,kw. | 159605 |
| 2 | ((“socioeconomic health*” adj3 difference*) or (avail* adj3 care) or (avail* adj3 healthcare) or (health* adj3 access*) or (unisur* adj3 health) or “access care” or “access healthcare” or crowding or “determinants of health” or diet or education or “education achieve*” or “education status” or employment or “environmental factors” or “financial difficult*” or “financial problem*” or “food insecurity” or “health literacy” or homeless or homelessness or housing or “housing instability” or Incarceration or “income difference” or indigent or “insurance health” or “insurance status” or “job insecurity” or jobless or “lack of educational attainment” or lifestyle or “low income” or marginalized or nutrition or “occupational status” or overcrowding or “physical activity” or poverty or “psychosocial depriv*” or “public safety” or “racial discrimination” or racism or “rural health” or SDH or SDOH or SES or “social and behavioral determinants of health” or “social behavior” or “social depriv*” or “social determinants” or “social determinants of behavior” or “social determinants of health” or “social disadvantage” or “social disparity” or “social economics” or “social environment” or “social exclu*” or “social factor*” or “social gradient*” or “social position” or “social support” or “social variation” or socialeconomics or socioeconomic or “socioeconomic circumst*” or “socioeconomic factor*” or “socioeconomic gradient*” or “socioeconomic position” or “socioeconomic status” or “socioeconomic status socioeconomic variable” or “standard living” or transportation or “underinsure* health” or underprivilege* or unemployed or unemployment or “vulnerable communit*” or “vulnerable group*” or “vulnerable people” or “vulnerable person*” or “vulnerable population*”).ti,ab,hw,kw. | 5462977 |
| 3 | (AI or “artificial intelligence” or “co-reference resolution” or “deep learning” or “information extraction” or “machine learning” or “named entity extraction” or “named entity recognition” or “natural language processing” or NLP or “predictive modeling” or “relation extraction” or “text mining”).ti,ab,hw,kw. | 269310 |
| 4 | 1 and 2 and 3 | 781 |
| 5 | limit 4 to english language | 779 |
| 6 | limit 5 to yr=”2010 -Current” | 751 |
| 7 | limit 6 to (letter or editorial or erratum or note or addresses or autobiography or bibliography or biography or blogs or comment or dictionary or directory or interactive tutorial or interview or lectures or legal cases or legislation or news or newspaper article or overall or patient education handout or periodical index or portraits or published erratum or video-audio media or webcasts) [Limit not valid in Embase,Ovid MEDLINE®,Ovid MEDLINE® Daily Update,Ovid MEDLINE® PubMed not MEDLINE,Ovid MEDLINE® In-Process,Ovid MEDLINE® Publisher; records were retained] | 61 |
| 8 | 6 not 7 | 690 |
| 9 | remove duplicates from 8 | 481 |

# Scopus

1 TITLE-ABS-KEY(EHR OR EHRs OR “electronic health record” OR “electronic health records” OR “electronic medical record” OR “electronic medical records” OR EMR OR EMRs)

2 TITLE-ABS-KEY((“socioeconomic health*” W/3 difference*) OR (avail* W/3 care) OR (avail* W/3 healthcare) OR (health* W/3 access*) OR (unisur* W/3 health) OR “access care” OR “access healthcare” OR crowding OR “determinants of health” OR diet OR education OR “education achieve*” OR “education status” OR employment OR “environmental factors” OR “financial difficult*” OR “financial problem*” OR “food insecurity” OR “health literacy” OR homeless OR homelessness OR housing OR “housing instability” OR Incarceration OR “income difference” OR indigent OR “insurance health” OR “insurance status” OR “job insecurity” OR jobless OR “lack of educational attainment” OR lifestyle OR “low income” OR marginalized OR nutrition OR “occupational status” OR overcrowding OR “physical activity” OR poverty OR “psychosocial depriv*” OR “public safety” OR “racial discrimination” OR racism OR “rural health” OR SDH OR SDOH OR SES OR “social and behavioral determinants of health” OR “social behavior” OR “social depriv*” OR “social determinants” OR “social determinants of behavior” OR “social determinants of health” OR “social disadvantage” OR “social disparity” OR “social economics” OR “social environment” OR “social exclu*” OR “social factor*” OR “social gradient*” OR “social position” OR “social support” OR “social variation” OR socialeconomics OR socioeconomic OR “socioeconomic circumst*” OR “socioeconomic factor*” OR “socioeconomic gradient*” OR “socioeconomic position” OR “socioeconomic status” OR “socioeconomic status socioeconomic variable” OR “standard living” OR transportation OR “underinsure* health” OR underprivilege* OR unemployed OR unemployment OR “vulnerable communit*” OR “vulnerable group*” OR “vulnerable people” OR “vulnerable person*” OR “vulnerable population*”)

3 TITLE-ABS-KEY(AI OR “artificial intelligence” OR “co-reference resolution” OR “deep learning” OR “information extraction” OR “machine learning” OR “named entity extraction” OR “named entity recognition” OR “natural language processing” OR NLP OR “predictive modeling” OR “relation extraction” OR “text mining”)

4 PUBYEAR AFT 2009 AND LANGUAGE(english)

5 1 and 2 and 3 and 4

6 DOCTYPE(le) OR DOCTYPE(ed) OR DOCTYPE(bk) OR DOCTYPE(er) OR DOCTYPE(no) OR DOCTYPE(sh)

7 5 and not 6

8 INDEX(embase) OR INDEX(medline) OR PMID(0* OR 1* OR 2* OR 3* OR 4* OR 5* OR 6* OR 7* OR 8* OR 9*)

9 7 and not 8

Web of Science

1. (TS=((EHR OR EHRs OR “electronic health record” OR “electronic health records” OR “electronic medical record” OR “electronic medical records” OR EMR OR EMRs) ) AND TS=(((“socioeconomic health*” NEAR/3 difference*) OR (avail* NEAR/3 care) OR (avail* NEAR/3 healthcare) OR (health* NEAR/3 access*) OR (unisur* NEAR/3 health) OR “access care” OR “access healthcare” OR crowding OR “determinants of health” OR diet OR education OR “education achieve*” OR “education status” OR employment OR “environmental factors” OR “financial difficult*” OR “financial problem*” OR “food insecurity” OR “health literacy” OR homeless OR homelessness OR housing OR “housing instability” OR Incarceration OR “income difference” OR indigent OR “insurance health” OR “insurance status” OR “job insecurity” OR jobless OR “lack of educational attainment” OR lifestyle OR “low income” OR marginalized OR nutrition OR “occupational status” OR overcrowding OR “physical activity” OR poverty OR “psychosocial depriv*” OR “public safety” OR “racial discrimination” OR racism OR “rural health” OR SDH OR SDOH OR SES OR “social and behavioral determinants of health” OR “social behavior” OR “social depriv*” OR “social determinants” OR “social determinants of behavior” OR “social determinants of health” OR “social disadvantage” OR “social disparity” OR “social economics” OR “social environment” OR “social exclu*” OR “social factor*” OR “social gradient*” OR “social position” OR “social support” OR “social variation” OR socialeconomics OR socioeconomic OR “socioeconomic circumst*” OR “socioeconomic factor*” OR “socioeconomic gradient*” OR “socioeconomic position” OR “socioeconomic status” OR “socioeconomic status socioeconomic variable” OR “standard living” OR transportation OR “underinsure* health” OR underprivilege* OR unemployed OR unemployment OR “vulnerable communit*” OR “vulnerable group*” OR “vulnerable people” OR “vulnerable person*” OR “vulnerable population*”)) AND TS=((AI OR “artificial intelligence” OR “co-reference resolution” OR “deep learning” OR “information extraction” OR “machine learning” OR “named entity extraction” OR “named entity recognition” OR “natural language processing” OR NLP OR “predictive modeling” OR “relation extraction” OR “text mining”))) AND LANGUAGE: (English) AND DOCUMENT TYPES: (Article OR Abstract of Published Item OR Data Paper OR Meeting Abstract OR Proceedings Paper OR Review OR Software Review) Indexes=SCI-EXPANDED, ESCI Timespan=2010-2020
2. PMID=(0*  or  1*  or  2*  or  3*  or  4*  or  5*  or  6*  or  7*  or  8*  or  9*)
3. 1 NOT 2

ACM Digital Library

Abstract:(EHR OR EHRs OR “electronic health record” OR “electronic health records” OR “electronic medical record” OR “electronic medical records” OR EMR OR EMRs)

AND

Abstract:(“access care” OR “access healthcare” OR “access to care” OR “access to health” OR “access to healthcare” OR “availability of care” OR “availability of healthcare” OR “available care” OR “available healthcare” OR “care availability” OR crowding OR “determinants of health” OR diet OR education OR “education achieve*” OR “education status” OR employment OR “environmental factors” OR “financial difficult*” OR “financial problem*” OR “food insecurity” OR “health access” OR “health insurance” OR “health literacy” OR “health uninsurance” OR “healthcare access” OR “healthcare availability” OR “healthcare uninsurance” OR homeless OR homelessness OR housing OR “housing instability” OR Incarceration OR “income difference” OR indigent OR “insurance status” OR “job insecurity” OR jobless OR “lack of educational attainment” OR lifestyle OR “low income” OR marginalized OR nutrition OR “occupational status” OR overcrowding OR “physical activity” OR poverty OR “psychosocial deprivation” OR “public safety” OR “racial discrimination” OR racism OR “rural health” OR SDH OR SDOH OR SES OR “social and behavioral determinants of health” OR “social behavior” OR “social behaviors” OR “social deprivation” OR “social determinants” OR “social determinants of behavior” OR “social determinants of health” OR “social disadvantage” OR “social disparity” OR “social economics” OR “social environment” OR “social exclusion” OR “social factor” OR “social factors” OR “social gradient” OR “social gradients” OR “social position” OR “social support” OR “social variation” OR socialeconomics OR socioeconomic OR “socioeconomic circumstance” OR “socioeconomic circumstances” OR “socioeconomic factor” OR “socioeconomic factors” OR “socioeconomic gradient” OR “socioeconomic gradients” OR “socioeconomic health difference” OR “socioeconomic health differences” OR “socioeconomic healthcare difference” OR “socioeconomic healthcare differences” OR “socioeconomic position” OR “socioeconomic status” OR “socioeconomic status socioeconomic variable” OR “standard living” OR transportation OR “underinsure* health” OR underprivilege OR underprivileged OR unemployed OR unemployment OR “uninsurance of health” OR “uninsurance of healthcare” OR “vulnerable communities” OR “vulnerable community” OR “vulnerable group” OR “vulnerable groups” OR “vulnerable people” OR “vulnerable person” OR “vulnerable persons” OR “vulnerable population” OR “vulnerable populations”)

AND

Abstract:(AI OR “artificial intelligence” OR “co-reference resolution” OR “deep learning” OR “information extraction” OR “machine learning” OR “named entity extraction” OR “named entity recognition” OR “natural language processing” OR NLP OR “predictive modeling” OR “relation extraction” OR “text mining”)**AND** [**Publication Date**: (01/01/2010 **TO** 04/08/2020)]

IEEE Xplore

(EHR OR EHRs OR “electronic health record” OR “electronic health records” OR “electronic medical record” OR “electronic medical records” OR EMR OR EMRs)

AND

(“access care” OR “access healthcare” OR “access to care” OR “access to health” OR “access to healthcare” OR “availability of care” OR “availability of healthcare” OR “available care” OR “available healthcare” OR “care availability” OR crowding OR “determinants of health” OR diet OR education OR “education achieve*” OR “education status” OR employment OR “environmental factors” OR “financial difficult*” OR “financial problem*” OR “food insecurity” OR “health access” OR “health insurance” OR “health literacy” OR “health uninsurance” OR “healthcare access” OR “healthcare availability” OR “healthcare uninsurance” OR homeless OR homelessness OR housing OR “housing instability” OR Incarceration OR “income difference” OR indigent OR “insurance status” OR “job insecurity” OR jobless OR “lack of educational attainment” OR lifestyle OR “low income” OR marginalized OR nutrition OR “occupational status” OR overcrowding OR “physical activity” OR poverty OR “psychosocial deprivation” OR “public safety” OR “racial discrimination” OR racism OR “rural health” OR SDH OR SDOH OR SES OR “social and behavioral determinants of health” OR “social behavior” OR “social behaviors” OR “social deprivation” OR “social determinants” OR “social determinants of behavior” OR “social determinants of health” OR “social disadvantage” OR “social disparity” OR “social economics” OR “social environment” OR “social exclusion” OR “social factor” OR “social factors” OR “social gradient” OR “social gradients” OR “social position” OR “social support” OR “social variation” OR socialeconomics OR socioeconomic OR “socioeconomic circumstance” OR “socioeconomic circumstances” OR “socioeconomic factor” OR “socioeconomic factors” OR “socioeconomic gradient” OR “socioeconomic gradients” OR “socioeconomic health difference” OR “socioeconomic health differences” OR “socioeconomic healthcare difference” OR “socioeconomic healthcare differences” OR “socioeconomic position” OR “socioeconomic status” OR “socioeconomic status socioeconomic variable” OR “standard living” OR transportation OR “underinsure* health” OR underprivilege OR underprivileged OR unemployed OR unemployment OR “uninsurance of health” OR “uninsurance of healthcare” OR “vulnerable communities” OR “vulnerable community” OR “vulnerable group” OR “vulnerable groups” OR “vulnerable people” OR “vulnerable person” OR “vulnerable persons” OR “vulnerable population” OR “vulnerable populations”)

AND

(AI OR “artificial intelligence” OR “co-reference resolution” OR “deep learning” OR “information extraction” OR “machine learning” OR “named entity extraction” OR “named entity recognition” OR “natural language processing” OR NLP OR “predictive modeling” OR “relation extraction” OR “text mining”)**AND** [**Publication Date**: (01/01/2010 **TO** 04/08/2020)]

**Supplemental Table 1.** Searching strategies used to retrieve the literature.

| **Category** | **Keywords** |
| --- | --- |
| EHR terms | electronic health record OR electronic health records OR electronic medical records OR electronic medical record OR EHR OR EMR |
| SBDH terms | socioeconomic health or (avail* adj3 care) or (avail* adj3 healthcare) or (health* adj3 access*) or (unisur* adj3 health) or “access care” or “access healthcare” or crowding or “determinants of health” or diet or education or “education achieve*” or “education status” or employment or “environmental factors” or “financial difficult*” or “financial problem*” or “food insecurity” or “health literacy” or homeless or homelessness or housing or “housing instability” or Incarceration or “income difference” or indigent or “insurance health” or “insurance status” or “job insecurity” or jobless or “lack of educational attainment” or lifestyle or “low income” or marginalized or nutrition or “occupational status” or overcrowding or “physical activity” or poverty or “psychosocial depriv*” or “public safety” or “racial discrimination” or racism or “rural health” or SDH or SDOH or SES or “social and behavioral determinants of health” or “social behavior” or “social depriv*” or “social determinants” or “social determinants of behavior” or “social determinants of health” or “social disadvantage” or “social disparity” or “social economics” or “social environment” or “social exclu*” or “social factor*” or “social gradient*” or “social position” or “social support” or “social variation” or socialeconomics or socioeconomic or “socioeconomic circumst*” or “socioeconomic factor*” or “socioeconomic gradient*” or “socioeconomic position” or “socioeconomic status” or “socioeconomic status socioeconomic variable” or “standard living” or transportation or “underinsure* health” or underprivilege* or unemployed or unemployment or “vulnerable communit*” or “vulnerable group*” or “vulnerable people” or “vulnerable person*” or “vulnerable population* |
| AI terms | NLP OR natural language processing OR information extraction OR named entity extraction OR named entity recognition OR co-reference resolution OR relation extraction OR text mining OR artificial intelligence OR machine learning OR deep learning OR predictive modeling OR AI |
